# Young Men’s Experiences of Being Fathered and Absent Father’s Experience: A Case Study from Urban Informal Settlements in South Africa

**DOI:** 10.1007/s43151-024-00118-1

**Published:** 2024-02-27

**Authors:** Smanga Mkhwanazi, Rachel Jewkes, Yandisa Sikweyiya, Laura Washington, Andrew Gibbs

**Affiliations:** 1 Gender and Health Research Unit, South African Medical Research Council, Durban, South Africa; 2 Project Empower (NGO), Durban, South Africa; 3 University of Exeter, The Queen’s Drive, Exeter, UK; 4 Centre for Rural Health, School of Nursing and Public Health, University of KwaZulu-Natal, Durban, South Africa

**Keywords:** Absent father, Young men, Emotions, Support, Social father

## Abstract

The impact of absent fathers can be a significant challenge for young people, but particularly for young men. Our study drew on 19 in-depth interviews with young men living in urban informal settlements in South Africa, to understand how they understood the impact of biological father absence. Young men described an idealized fatherhood role in which biological fathers provided economic support, active fathering (including emotional support), and social recognition of children. Young men described biological father absence in very emotional terms, including the exclusion from family networks, and having negative economic and educational impacts. Furthermore, men saw biological father absence as impacting on their current situation, and as part and parcel of their wider social marginalization in South Africa. Social fathers — alternative male role models as they grew up — while described as existing, were not felt to be adequate in replacing biological fathers, despite the economic support and guidance some described receiving. Engaging with young men around the repercussions of biological father absence is important for supporting young, poor men in South Africa.

## Background

South Africa is a society in which traditionally descent is determined through the male line and is validated by complex marriage practices (Parker 2015) and where marriage rates are dramatically declining, especially among younger African people. Marriage rates among young (20–30 years old) African women declined from just under 40% in 1995 to 24% in 2008, while among White women, there was only a small reduction (just over 70 to 67%) in the same time period (Posel and Rudwick 2013). Marriage rates also vary by province, in KwaZulu-Natal (where this study is based). Black women aged 20–30 years have much lower rates of marriage than national averages (Posel and Rudwick 2013). The racial differences in marriage rates are clearly driven by the impact of apartheid and how circular migration impacted on families (Swartz 2009). In this context, approximately two-thirds (64%) of children grow up without their biological father present in their household although 35% of these children co-reside with another adult male.

The experiences of fatherhood as well as father presence and absence in South Africa have been historically influenced by apartheid and its legacy (Morrell 2006, Richter 2006), and shaped how many South African men related to their children (Chauke and Khunou 2014, Morrell 2006, Spjeldnaes et al. 2011). The South African apartheid system, based on racial segregation, forced men to work in mines far from their homes and spend little or no time with their children (Bhana and Nkani 2014; Ratele et al. 2012; Spjeldnaes et al. 2011), and men’s relationship to their children was further worsened by the HIV/AIDS pandemic that left many young people with no fathers (Ratele et al. 2012). Today in South Africa, these issues have been exacerbated by unemployment, poverty, and global economic challenges (Morrell 2006), which when intersecting with norms of masculinity has meant that father absence can be a common experience. 

Alongside the structural drivers shaping father absence, there are a range of locally determined social practices that also impact on father absence. A variety of social practices, such as lobola (bride wealth) and “damages” for making a woman pregnant outside of marriage, still play a role (at times) in shaping men’s relationships to their children (Makusha and Richter 2016; Kaufman et al. 2001). This is particularly the case among men who have little or no access to work, and therefore struggle to access money to pay these costs and are therefore sometimes excluded from accessing their children (Makusha and Richter 2016; Kaufman et al. 2001).

Evidence suggests that children who grow up without biological fathers present in their lives have worse health, wellbeing, and economic outcomes than children whose fathers are present (Rosenberg and Wilcox 2006). For instance, a twenty-one-country study, including South Africa, found children with absent fathers had more behavioral problems and were less likely to complete school (Freeks 2017). Another study in South Africa found that children who grew up without their biological father’s involvement in their educational development had poorer mental health and learning outcomes and were more vulnerable to dropping out of school (Salami and Okeke 2018). A further study conducted in South Africa (Langa 2020) among young men found that they connected father absence to smoking and drinking alcohol at a young age.

While considerable research has focused on the underlying causes of absent fathers in South Africa (Engle and Breaux 1998, Morrell et al. 2016, Martinez et al. 2013), much less research has examined children’s and young adults’ emotions and feelings about their absent biological fathers, particularly focusing on boys and young men as they grow up and mature into adulthood. While few, those studies that have explored this question have described young men and boys feeling “pain” about not knowing their biological father, which manifested in a “crisis of identity,” and being envious of friends with present biological fathers (Langa 2020, Pitsoane 2014). Others have highlighted how young men felt they did not have the necessary economic and emotional support they needed to make progress in life because of father absence (Mavungu 2013, Morrell et al. 2016). Similarly, Langa (2020) outlines how some young men describe feelings of depression because of not having their biological father in their life.

Other research has pushed back strongly against the idealization of the role of the biological father, emphasizing how other adult men, termed “social fathers,” do play an important role in children’s development in South Africa (Kesebonye and Amone-P’Olak 2021; Ratele et al. 2012). Social fathers refer to either other male family members, or community members, who play a role in socializing and supporting children when biological fathers are absent (De Boise and Hearn 2017, Lomas 2013). While research on social fathers is limited, studies describe social fathers as playing an important role economically supporting children and to some extent providing care and guidance (Clowes et al. 2013; Malherbe and Kaminer 2022) and becoming “role models” for boys and young men. While there is much positive literature on the potential role of social fathers in young men’s lives, there remains a persistent underlying view from boys and young men that biological fathers remain an important (missing) part of their lives (Clowes et al. 2013; Malherbe and Kaminer 2022).

Understanding how boys and young men create expectations of biological fathers and social fathers and the impact of absent biological fathers requires understanding how fatherhood is constructed within specific local understandings of men and masculinities (Ratele et al. 2012). In any given context, there is an idealized form of masculinity, which is typically unattainable (Dolan and Coe 2011), which includes a framing of what fathers are and should do (Osborne et al. 2014). In South Africa, there has been substantial discussion focused on men and masculinity and how fathers and fatherhood intersect, shape, and are shaped by wider notions of “masculinity” (Chauke and Khunou 2014; Denis and Ntsimane 2006, Lindegger 2006, Morrell 2006, Richter 2006). A central component of the idealized father identity is a father’s role of economic provision, which Helman et al. (2019) suggest, has become part of the “hegemonic” marker of masculinity in the context of the father’s role (Helman et al. 2019). Other aspects of successful fatherhood may well include being able to engage in cultural rites of passage including *ukubikwa, inhlawulo*, and *ukufakwa isiphandla*, as well as playing a guiding role in the child’s development (Malinga 2015). *Ukubikwa* refers to the traditional ceremony where the child is introduced to the biological family, and sometimes this may include the ancestral ceremonial performance, while *inhlawulo* includes paying for damages, and this signifies that the biological father accepts the child and responsibility. U*kufakwa isiphandla* is an ancestral ceremonial performed to recognize and accept the child in the family. Increasingly, there are also expectations of active fathering (such as playing with the child) as being central to successful fatherhood (Athour-1 et al. 2017). Importantly, the emergence of research on queer families (e.g. Patterson 2000) and adoption has required consideration of how different roles and identities may be constructed and how research on heterosexual families implicitly reifies these relationships (Manalansan IV, 2006, Patterson 2000). These different concepts of successful fatherhood are often overlapping and difficult to disentangle, and indeed, it may be that only through achieving it all can successful fatherhood be achieved. In this way, expectations of successful fatherhood are social, rather than biological, construct, as successful fathers need to achieve some (if not all) of these different components.

In this paper, we seek to reorient the focus of the research around fathers and fatherhood towards how young men who have often experienced growing up without biological fathers understand this impact on them and how this shapes their sense of their position in society. We seek to move beyond a focus only on the economic impacts of father absence to include a focus on emotional impacts of absent fathers, and how men sought to establish social fathers as a form of having a father figure in their lives. We use qualitative data collected from young (ages 18–30 years) men living in urban informal settlements, around eThekwini Municipality, South Africa.

## Methodology

We conducted a secondary analysis of qualitative data — in-depth interviews — collected among 19 young men involved in the Stepping Stones and Creating Futures (SSCF) intervention project in urban informal settlements in eThekwini Municipality, South Africa, which were collected to understand the impact of [redacted for blind review] intervention. Urban informal settlements are marked by lack of resources, high unemployment and poverty, and violence (Meth 2017). Moreover, urban informal settlements experience numerous challenges ranging from high levels of community violence, unemployment, and limited social support, leading to high rates of violence, alcohol misuse, and poor mental health (Athour-2 et al. 2018). The SSCF intervention aims to lessen young men’s perpetration of Intimate Partner Violence (IPV), and strengthen livelihoods, and was delivered by trained peer facilitators over 3 months, to small groups of young men (Athour-3 et al. 2020).

As part of a qualitative process evaluation to understand pathways and barriers to change in the intervention (Athour-3 et al. 2020), we identified two clusters (comprising ~ 40 men) from the 17 clusters receiving the intervention. Clusters were identified based on ease and safety of access for the research team. All young men in the two clusters were invited to participate in the study (*n* = 40), but only 19 agreed. The 19 young men who agreed were interviewed at baseline, and we attempted to repeat interviews with them at 12 and 18 months. We conducted repeated interviews to understand men’s experiences of the intervention and how the intervention may have impacted on their lives, with data collected at baseline, 12 months and 18 months. In addition, we also conducted “light-touch” observations with men in one cluster to better understand their daily lives and experiences and add depth to the interviews. Given the high mobility of youth searching for employment opportunities in this setting, we found that some men had moved out of communities to search for work.

At baseline, interviews focused on young men’s lives, families, and livelihoods, as well as relationships and perpetration of violence. At 12 and 18 months, we asked similar questions, but also focused on their subjective experiences of involvement in the intervention and how they felt things had changed. In none of the interviews did we ask directly about men’s fathers, nor their fathers’ involvement in their childhood, though we did ask about their experiences growing up at baseline. Also, we did not ask participants about their role as a father to their children, rather only asked if they have children. Our lack of direct questioning was because our intervention was not designed to tackle issues and experiences of fathers and fatherhood. Our qualitative interviews were conducted in isiZulu by two trained research assistants and lasted between 45 min and 1.5 h and were digitally recorded. Data were translated and transcribed into English.

We undertook a secondary analysis of the qualitative data using Braun and Clarke’s (2006) thematic analysis. This approach to thematic analysis allowed us to be flexible with the data (Braun and Clarke 2006) and provide a complex and rich set of accounts. We started by reading all the transcripts to understand young men’s experiences of being fathered. We then wrote summaries of each transcript focusing on men’s descriptions of being fathered (or not) and then conducted open thematic coding on the whole dataset identifying small codes related to their fathers. We then sorted these small codes into sub-themes and main themes. We then organized these main themes to understand the relationship between the different codes. We present these themes below in the results section.

The study received ethical approval from the [blinded for peer review], and [blinded for peer review]. All men provided written informed consent for the main trial, and separately for the qualitative study. We used pseudonyms for participants and ensured identifying information was removed.

## Results

In total, nineteen men completed at least one interview (Table 1). At baseline, most men were not working in formal employment, only two were studying, and those who did work were doing temporary work (piece jobs). One man lived with his father and described his father as supportive; five reported that they grew up with their father, but did not live with him now; and thirteen (of nineteen) reported that they grew up without their biological father present. Some of the thirteen reported having tried to establish a relationship with their father, but none reported success. Among those who had grown up with their fathers, most reported still maintaining contact.

**Table 1:** Participants socio-demographic information and relationship to father

Name and age (at baseline)	Currently working (yes/no)	Highest education	Relationship to biological father (yes/no)	Baseline interview (yes/no)	First follow-up interviews	Second follow-up interviews	Household dynamics /living with social father
Bafo, 26 years	Yes	Completed high school	No—he doesn’t have a good relationship with his father	Yes	Yes	No	No—lives with a mother and his sister
Jeqe, 20 years	No	Completed high school	No—never met his father since birth	Yes	Yes	Yes	No
Kay 2, 21 years	No	Grade 10	No—his father has married someone else	Yes	Yes	No	He lives with his uncle
Kehla, 22 years	No	-	Yes—has a good relationship with his father	Yes	No	No	No social father
Lihle, 20 years	Yes	Completed high school	Yes—Lihle’s father died in 2010	Yes	Yes	Yes	Lives with older brother
Matha, 24 years	No	Tertiary -in progress	Yes—mother passed on—they were living as a family	Yes	Yes	Yes	No
Njabulo, 26 years	Yes	Completed high school	No—he saw his father for the first time in 2002 and in 2003, he died	Yes	No	No	He lives with his mother and his younger brother
Nsimbi, 24 years	Yes	Final year of high school, but not passed	No—he knew his father recently and feel that he doesn’t trust his father	Yes	Yes	Yes	No—he lives with his friends
Sam, 27 years	Yes	Completed high school	No—Sam lives with his mother and stepfather	Yes	Yes	Yes	Yes
Sanele, 27 years	Yes	Grade 10	No—father died, he cheated and separated with the mother	Yes	No	No	No
Sbo, 23 years	Yes	Completed high school	No—father died while doing grade 4	Yes	Yes	No	No
Scelo, 22 years	Yes	Final year of high school, but not completed	No—his father passed on	Yes	No	No	Scelo relies on his friend for a living
Sphiwe, 23 years	No	Drop out at high school	Yes—he had good relationship with his father before he passed on	Yes	No	No	-
Thabiso, 21 years	No	Grade 10	No—his father is married to someone else	Yes	No	No	Live with his uncle
Thula, 21 years	No	Grade 7	No—his father is alive, but they don’t communicate	Yes	Yes	Yes	Lives with his three brothers, but next door father assists him
Menzi, 22 years	No	Unknown	No—he doesn’t have a good relationship	Yes	No	No	Lives with aunt and older brother
Welile, 22 years	Yes	Completed high school	Yes—he still communicates, and they have a good relationship	Yes	Yes	Yes	Lives with older brother
Xolani, 26 years	Yes	High school dropout	No—father died due to political violence	Yes	Yes	No	He lives with grandparents
Nhlanhla, 26 years	Yes	Completed high school	No—his father fights with everyone in the family	Yes	Yes	No	Aunt and his husband support him

Across the interviews, definitions of successful masculinity included being a father, and being able to provide economically for a family, with the implicit assumption of a wife and children. Through achieving these markers, a successful man was someone looked up to, especially by young men, in the community and was respected by others:

Lihle (20 years) …as I mentioned earlier, a man is a father with a house, children and he is the one who looks after the household. Like that… a man is a person who is able to take care of his family.

Successful fatherhood extended far beyond the biological conception of a child and simple provision to a family, but also included the ways in which a father should behave in relation to family and the broader community:

Jeqe (20 years)…as a respected father it makes your house a respected one, people also like you if ever you are in trouble, they are able to assist you.

Young men saw the central role of fathers as providing what they termed “guidance” and wider social support rather than simply economic support:

Bafo (26 years) every house is a house because of a man, a man should play his role at home, when kids grow, they need help from their dad, a man is the one that carries a family.

Yet, despite all the young men who were interviewed having very clear understandings about what fathers *should* do in relation to establishing households and raising their children, the realities they described in their own relationships with their biological fathers were more complicated. Indeed, although we did not ask them directly about their biological fathers, they often spoke extensively about them, primarily in terms of them being absent, but also present, in their lives. Broadly, two forms of support (or absence) by their biological fathers were described by young men, the first was emotional support, the second being material support, particularly for education, but also daily life. Men also reflected on the impact of their biological fathers on how they now lived their lives. Where biological fathers were absent, they also discussed how they sought support from men in the community that they understood as father figures and prominent figures in the community, such as local businessmen, pastors, and family men.

Many young men spoke extensively about the emotional impact of not having a biological father as they grew up, often describing it in terms of a “missing-ness,” or a gap, in their life:

Interviewer: Is there anyone you would say you see the need of them, but they are not present in your life?

Nhlanhla (26 years) Ey bro I would say it’s my father, you see the absence of my father is a problem. Because there are a lot of things that would probably happen. My father was educated back in the day maybe even my life wouldn’t be as it is right now. There are a lot of things maybe he would do for me, even his teachings I don’t know them, so I would be the person…I would say in my life there is a void somewhere.

Sbongiseni referred to a “gap” in his life because his biological father was not around as he grew up, and spoke about how his life could have turned out differently if he had been around:

Sbongiseni (23 years) Maybe I would have needed my father for that with regards to certain things I am not supposed to do or I am supposed to do, which is a father’s role that he is meant to play in his child’s life. …There is that gap which I feel he was supposed to be there, but he wasn’t there to assist me with things like that. Maybe my life would be different, perhaps I wouldn’t be living this life.

The young men who had grown up with their biological father present spoke primarily about the emotional support and care provided by them, rather than the material support that their father provided. Matha, for instance, described how after his mother died, his biological father played a central role in raising him and caring for him in ways that he felt a mother would:

Matha (24 years) let me see, the time when mom passed away, he was with our family he showed us that he is a father to us and he showed us love, he also loves mom, it has been 13 years since mom passed but he has never brought a woman home, if he does, he does it far from here. Secondly when I was in matric I became very sick, when I was sick he played a role of a mother to me, he took care of me a lot… : because he knows that I respect him, I also love him, in everything that comes up be it a job or a problem I tell him. …most of the time we watch soccer, we talk, we also like the same sport.

Feeling cared for and loved by their biological father was seen by participants as a vital component in their upbringing, with many young men speaking about how they appreciated the effort made by their fathers to provide care for them:

Xolani (26 years) when Mom and Dad were still alive they used to love me a lot, they knew that I know how to respect elders…

In contrast, young men who grew up without biological fathers often described feeling unloved and disowned by this absence, often in terms of lack of material support:

Nsimbi (24 years) …he [my father] doesn’t want to do the right thing and support me…

Young men also spoke about how biological fathers were important for them in having someone to talk to, listen to the challenges they faced growing up, and provide advice. Where biological fathers were absent, the young men felt they had few people to guide them on the challenges of growing up:

Sbongiseni (23 years) …You yourself know that when a young boy or girl goes into high school, the family normally gives advice and guidance about life. So now there is no one who has ever sat me down as I am a male and said if you are at this stage in life these are the things you must do. Maybe I would have needed my father for that with regards to certain things I am not supposed to do or I am supposed to do, which is a father’s role that he is meant to play in his child’s life.

Men also described how biological fathers were important to help them become “young men,” and without a senior adult man to guide them, they felt adrift:

Sbongiseni (23 years) My father is not here, he could have taught me how to conduct myself as a man, but he didn’t, and thus I do not know how to conduct myself.

It may be that these young men are describing a general sense of lack of male guidance in their lives and in their families as they grew up. It alternatively could be that this was describing their feeling of exclusion from family rituals, of the absence of cultural processes, the equivalent of which would be the advice given at times of different family ceremonies and rituals, as to how they carry family forward and carry themselves as a man.

Some of the young men who had not had biological fathers growing up also felt lost and cut off from their paternal family as they could not use their father’s surname. Among these young men, there was a strong feeling that biological fathers needed to perform cultural customs such as *ukubikwa* and *ukufakwa isiphandla.* These traditional ceremonies performed for a child welcomed the child into the family and allowed the child to use his father’s surname, and to be protected by ancestors. For the men, the symbolism of being able to use their biological fathers’ surname was that they would then feel “complete” as men, as well as believing they would be given guidance and support by their father, as providing them with luck around navigating life:

Xolani (26 years) I want to use my [Father’s] surname but I won’t use the Ndlovu [Father’s] surname. But, I will use a surname that I will create for myself because I have problems when I talk about this surname [Msomi] because my father did not do anything for me, he did not show up [to be active and present in Xolani’s life], maybe he is alive? You know that I don’t know my father it is the truth that I’m telling. I don’t know my father since day one I was born.

Among men who described their biological father being absent as they grew up, almost all also talked about the economic impact of this. Several participants spoke about how they struggled to continue with school because they did not have school necessities such as uniform and schoolbooks due to a lack of support from their fathers:

Jeqe (20 years) I don’t think [I need my Father’s support now] so because I am grown now. I needed him when I was still in the lower grades because Mom was struggling. She had to buy me school stuff and so on, that’s when I needed him the most, but now it’s fine, he may not appear… for him to support Mom, school fees, school books.

Participants with absent biological fathers also described how they felt their progress in school was limited as their father could not help them with homework. Yet among those with biological fathers in their lives as they grew up, there was no specific evidence that fathers had supported them with their homework. This idealization of “what could have been” was prominent in stories of absent biological fathers, and contrasted often with men’s experiences of present biological fathers, as Thabiso shows with his own father’s limited interest in schooling:

Thabiso (21 years) I would have been far (ahead) with school, I would have even started school at an early stage. … I continuously dropped-out of school, so if my mother was there, we would have stayed home and as her child she would have sent me to school.

Men who had not been economically supported by their biological fathers described not just material impacts, but also profound emotional implications: Thula, 21 years “…because my father, it’s like he doesn’t exist even though he is alive because he does not help us with anything”*.* Similarly, Menzi described his frustration and disappointment that his biological father was not willing to support him, contradicting what fathers should do:

Menzi (22 years) …Ayi that man [his Father]! You don’t even know what kind of a man he is. If you speak to him or seek advice from him, he will still do that thing. If you ask him for money, he will hide by saying he doesn’t have any money. But you can see that he does have money.

In the absence of biological fathers, many participants discussed the importance of having another adult male to help them navigate life, “social fathers” ranging from stepfathers, friends, and extended family members:

Sam (27 years) …yes there is my stepfather, it is like he was the one that raised me from the time I was young till now

Thula described how in the neighborhood he had a “social father” who helped him in times of need, was someone he could talk to, and one who played a father role in his life.

Thula (21 years) There’s another father that I’m close to him, if I need something, he does help me, and he doesn’t look at people as low. Most of the time he chats with me and takes me as his own child, sometimes he takes me out and buys me something that I like.

Despite the potential role of social fathers in providing economic and social support to some young men, there remained a sense that biological fathers could and would go further to provide care for their children and this was almost intuitive. For Matha, who lived with his father, he described this as: “he feels what is needed from him is to show love and be part of his family; he also needs to support his family in any way possible.” Similarly, Nsimbi (24 years) although not knowing his father until recently suggested and almost biological connection between himself and his father:

Nsimbi (24) me… you see, somethings just happen you see, I don’t want to go and tell him where I am coming from or where I am going with life but for him as a parent… every parent knows their child or where they want to go from where they are.

## Discussion

In this paper, we explored young men’s experiences of fathers and especially the perceived impacts of the absence of biological fathers, which builds on and extends much South African research that has looked at the experiences of being a father (Hunter 2019, Morrell 2006, Posel and Devey 2006, Athour-4 et al. 2022). Our analysis went further to try and understand the social, psychological, emotional, and economic impacts of biological father absence and how it impacted on young men’s sense of identity. Among young men living in urban informal settlements in Durban, our analysis highlighted how men held strong views about what biological fathers should do, which emphasized a patriarchal masculinity, comprising of three interlocked roles: economic provision to the family and child, active fathering (including emotional care and support), and finally, social acknowledgement (recognition of the child through various public/family events). These roles of a successful father were idealized by men, yet many experienced a significant disjuncture between these ideals and their own experiences of being fathered. They described how it impacted on them emotionally and socially, and broadly felt it reinforced their wider sense of failure they felt about their overall position in life, thus strengthening their sense of being socially marginalized as a group. Indeed, some young men mentioned a lack of pride and confidence as a result of absent biological father, and saw this as connected to a lack of emotional relationships with their own children.

Previous research has also sought to describe dominant assumptions about idealized fathers in South Africa (e.g. Gardiner 2004) with much research emphasizing the importance of economic provision (Langa 2020, Ratele et al. 2012, Helman 2015), and the challenges that young men in particular face in meeting this in the context of the legacies of apartheid and neo-liberal/post-capitalism (Holborn and Eddy 2011). In addition, young men were also clear that a successful masculinity/fatherhood should include providing emotional support, guidance, and care to children, which may be indicative of a shift in expectations of fatherhood towards an active fatherhood. This shift towards more “caring” masculinities may be part and parcel of a wider shift being seen globally with social norms of men being engaged fathers in children’s emotional care, as well as economic providers (Holborn and Eddy 2011), and a movement towards more gender equitable relationships. Finally, young men also identified the importance of social acknowledgement by their father, often through public acknowledgement in family and community events, and through drawing on cultural practices. Also, some young men mentioned the importance of being identified by their fathers in the community as a crucial marker for their identity and they believed that this aligned them with their heritage (Langa 2014).

Together these three components, economic provision, active fathering, and social acknowledgement, comprised a dominant understanding of successful father masculinity, and were interlinked and contingent on one another. While there were evolving norms of what roles biological fathers *should* play in their children’s lives, the majority of men described experiences where these roles had not been met as they grew up. Those men without engaged biological fathers described how they felt there were economic impacts of biological father absence as they grew up, including on school progression, and studies have described similar findings. In this study, it was also evident that biological fathers’ economic failings also translated into an emotional impact for young men, as they saw the lack of economic provision as indicative of their biological father’s lack of love for them. Previous research in similar settings has described how among heterosexual adults, economic provision is a demonstration of love (Hunter 2019, Rosenberg and Wilcox 2006), and this pattern seemingly extended to parent–child relationships. The failure of biological fathers to provide was clearly perceived as a violation of norms.

Importantly, young men were also concerned about how biological father absence meant they were not socially acknowledged through a range of “customary” ceremonies and processes and the impact this had on them emotionally and in terms of their identity. Previous research has identified how biological fathers often end up not being actively involved in children’s lives because of failing to meet cultural obligations, such as paying lobola (Makusha and Richter 2016), because they cannot afford it. Indeed, Richter et al. (2010) illustrate how “customary law” negatively impacts on fathers’ participation in childhood development. Yet, little, if any, research has considered the impact on young men excluded from cultural processes and rituals due to biological father absence. Young men were unable to participate in traditional ceremonies, nor take their biological father’s surname (Langa 2010), and felt excluded from an important cultural aspect of their life. In this context, the importance of passing on of the surname is important for both the father and the child, conveying recognition and incorporation into a family (Engle and Breaux 1998, Athour-1 et al. 2017, Helman 2015). For these young men, becoming a “man” was a cultural and social process, not simply an age-related process.

Although not clearly articulated in these narratives of father absence, the impact of the absence and the emotional, economic, and cultural implications of this, further reinforced young men’s sense of marginalization and of being “children.” In previous work, young men in marginalized settings, without access to economic, social. and political resources, have described feeling as if they were “children” because they could not work and establish homes (Engle and Breaux 1998, Athour-1 et al. 2017, Helman 2015). Biological father absence further enhanced young men’s feelings of exclusion and marginalization. Some of this was linked to practical exclusions, as they felt a biological father would have supported them through education or into work, while other aspects were around feeling bereft of “guidance” around growing up and making sense of their life, and feeling disconnected from biological patriarchal families because of not being formally acknowledged. These exclusions reinforced young men’s overarching sense of being outside the mainstream.

A few of the young men mentioned other adult men who had played or continued to play the role of “social fathers” in their lives, and young men were actively seeking to construct alternative forms of relationships to “fathers.” A number of other studies have similarly described “social fathers,” who can include men from the extended family, and the community including soccer coaches, and teachers (Coakley 2006, Langa 2020, Athour-4 et al. 2022). These social fathers can play an important active role in children’s economic and emotional lives (Clowes et al. 2013; Malherbe and Kaminer 2022).

Despite the promise of social fathers in offering a way to buffer against the impact of the absence of biological fathers, young men typically did not see social fathers as being an adequate replacement and continued to idealize the concept of an engaged biological father. Other research has similarly highlighted that while social fathers can play an important role, they are still seen as secondary to biological fathers (Athour-4 et al. 2022). It is unclear why this may be, but it may be linked to the dominant patriarchal notions of biological family, as well as the exclusion from a range of cultural practices.

Our study had several limitations. The study was not designed to assess the impact and experiences of young men with absent biological fathers, yet despite us not directly asking about these issues, it came up in young men’s own stories and narratives, with young men repeatedly reporting their experiences and impact of living without their father, suggesting it was a significant issue for them. We also did not specifically ask about social fathers and so cannot be sure the extent to which they were, or were not, important in young men’s lives or whether they could adequately replace biological fathers. We also had a limited sample and did not probe about the queer parenting or families, with young men only speaking of heterosexual families; further research on how queer parenting may shape this experience is needed. In addition, we only interviewed young men from two informal settlements, and as such, we cannot generalize our study results to the wider population. We have sought to enhance transferability through providing detailed descriptions of young men’s experiences and the context they live in. It would be important to undertake focused research on father absence among young women living in urban informal settlements to understand and compare their experiences. Importantly, the first author conducted half of the interviews and supported the interpretation of local concepts and meanings.

## Conclusion

Young men, growing up in the informal settlements, defined successful fathers’ role in three aspects: economic provision, active fathering, and social acknowledgement. Living in challenging circumstances, their expectations of how they should have been fathered were shaped strongly by local conceptualizations of what constituted a successful biological fatherhood, and they interpreted their own experiences of their fathers, whether present or absent, through this idealized notion. Given that the idealized form of biological fatherhood was rarely, if ever, experienced because of the long legacies of apartheid, poverty, the decline of marriage, and changing gender norms, young men suffered because their childhood experiences did not live up to the ‘”ideal” experience. This threatened their subjective ability to assume manhood both socially and in the metaphysical patrilineage. This contributed to their overall sense of exclusion in life, as young, unemployed, poor men living in urban informal settlements. While some men did describe social fathers as being important, social fathers could not adequately replace biological fathers in these young men’s lives, limiting the potential of social fathers to support young men.

This analysis opens several important spaces to support young men through work focused on deconstructing the meaning of fatherhood. First is to recognize the important of role of emotional and social engagement of biological and social fathers in children’s lives, beyond economic provision. In developing interventions, this could be an important area to work with in strengthening relationships, as it is something achievable without financial resources and was important for young men. Second, social fathers were apparent in men’s lives. While these relationships were of reduced symbolic value, they still provide an entry point through which through young men’s needs for economic support and emotional guidance could be addressed. Understanding how biological father absence impacted on young men’s construction of masculine identities is an important first step in working to support the development of alternative identities that are more embracing of diversity. The data used in this study can be made available upon request.
